# Development and evaluation of a mobile application for case management of small and sick newborns in Bangladesh

**DOI:** 10.1186/s12911-019-0835-7

**Published:** 2019-06-20

**Authors:** Lauren E. Schaeffer, Salahuddin Ahmed, Mahmoodur Rahman, Rachel Whelan, Sayedur Rahman, Arunangshu Dutta Roy, Tanzia Ahmed Nijhum, Nazmun Nahar Bably, Helen D’Couto, Carly Hudelson, Iffat Ara Jaben, Sayed Rubayet, Abdullah Baqui, Anne CC Lee

**Affiliations:** 1 Department of Pediatric Newborn Medicine, Brigham and Women’s Hospital, Suite BB-502, 75 Francis Street, Boston, MA 02115 USA; 2 Projahnmo Research Group, Johns Hopkins University- Bangladesh, “Abanti”, Flat #5B, House #37, Road #27, Banani, Dhaka, 1213 Bangladesh; 3 Maternal and Child Health Division, International Centre for Diarrhoeal Disease Research, Bangladesh, 68, Shaheed Tajuddin Ahmed Sarani, Mokakhali, Dhaka, 1212 Bangladesh; 4 Health Section, United Nations Children’s Fund, Rangpur Field Office, Rabdhaballav Road, Rangpur, 5400 Bangladesh; 5 Harvard Medical School, 25 Shattuck St, Boston, MA 02115 USA; 6 Save the Children: Bangladesh, House CWN (A) 35, Road 43, Dhaka, 1212 Bangladesh; 7 Johns Hopkins Bloomberg School of Public Health, 615 N Wolfe St, Baltimore, MD 21205 USA

**Keywords:** m-Health, Newborn care, Newborn case management, Newborn assessment, User-centered design, Integrated Management of Childhood Illnesses, mCNCP, Comprehensive Newborn Care Package, Community health worker, Bangladesh clinical guidelines, Newborn danger signs

## Abstract

**Background:**

In low-income settings, community health workers (CHWs) are frequently the first point of contact for newborns. Mobile technology may aid health workers in classifying illness and providing referral and management guidance for newborn care. This study evaluates the potential for mobile health technology to improve diagnosis and case management of newborns in Bangladesh.

**Methods:**

A mobile application based on Bangladesh’s Comprehensive Newborn Care Package national guidelines (mCNCP) was developed to aid CHWs in identifying and managing small and sick infants. After a 2-day training, CHWs assessed newborns at Sylhet Osmani Medical College Hospital and in the Projahnmo research site (Sylhet, Bangladesh) using either mCNCP or a comparable paper form (pCNCP), similar to standard IMCI-formatted paper forms. CHWs were randomized to conduct a block of ~ 6 newborn assessments starting with either mCNCP or pCNCP, then switched to the alternate method. Physicians using mCNCP served as gold standard assessors. CHW performance with mCNCP and pCNCP were compared using chi-squared tests of independence for equality of proportions, and logistic regressions clustered by CHW.

**Results:**

Two hundred seven total CHW assessments were completed on 101 enrolled infants. mCNCP assessments were more often fully completed and completed faster than pCNCP assessments (100% vs 23.8%, *p* < 0.001; 17.5 vs 23.6 min; *p* < 0.001). mCNCP facilitated calculations of respiratory rate, temperature, and gestational age. CHWs using mCNCP were more likely to identify small newborns (Odds Ratio (OR): 20.8, Confidence Interval (CI): (7.1, 60.8), *p* < 0.001), and to correctly classify 7 out of 16 newborn conditions evaluated, including severe weight loss (OR: 13.1, CI: (4.6, 37.5), *p* < 0.001), poor movement (OR: 6.6, CI: (2.3, 19.3), *p* = 0.001), hypothermia (OR: 14.9, CI: (2.7, 82.2), *p* = 0.002), and feeding intolerance (OR: 2.1, CI: (1.3, 3.3), *p* = 0.003). CHWs with mCNCP were more likely to provide counseling as needed on 4 out of 7 case management recommendations evaluated, including kangaroo mother care.

**Conclusions:**

CHWs in rural Bangladesh with limited experience using tablets successfully used a mobile application for neonatal assessment after a two-day training. mCNCP may aid frontline health workers in Bangladesh to improve completion of neonatal assessment, classification of illnesses, and adherence to neonatal management guidelines.

**Electronic supplementary material:**

The online version of this article (10.1186/s12911-019-0835-7) contains supplementary material, which is available to authorized users.

## Background

Identification and management of neonatal illnesses are challenging in low- and middle- income countries (LMICs), where human resources are limited and health systems may be weak. Over half of births in LMICs occur at home, and community-based health workers (CHWs) are often the first to assess these newborns [1]. CHWs typically have multiple tasks with inadequate time to complete the newborn assessment, and may have limited clinical knowledge for complex calculations or decision-making. Identifying and managing preterm (< 37 weeks of gestation) and small-for-gestational age (SGA; < 10% birth weight for gestational age) infants, who have increased risks for morbidity and mortality, present their own challenges in LMICs [2–4]. Gestational age calculation requires a last menstrual period (LMP), that is often missing or requires computation. SGA determination requires a weight-for-age percentile calculation or standard chart reference [5, 6]. Furthermore, small and preterm infants have special clinical needs, including extra warmth and feeding support [7], and may benefit from earlier referral and more frequent follow-up visits to support their survival and development [8, 9]. Clinical decisions about case-management (such as when to refer or specific antibiotic dosing) are often dependent on infant size, gestational age, or postnatal age, and can be cumbersome for CHWs.

These aforementioned challenges are present in Bangladesh, where 62% of births occur outside the home [10], and CHWs are usually the first to assess newborns within the first days of life [10]. The neonatal mortality rate is high at 28/1000 live births [10]. The majority of neonatal deaths occur among preterm or SGA infants [11], and prevalence of preterm birth is 22.3% and SGA is 39.6% [12, 13]. Many of these deaths can be prevented with interventions if newborn danger signs are identified and managed early [3]. In 2013, Bangladesh’s Ministry of Health and Family Welfare (MOHFW) introduced the Comprehensive Newborn Care Package (CNCP) national training and implementation guidelines for newborn care [14, 15], with an emphasis to reduce neonatal mortality through evidence-based interventions, such as chlorhexidine application for umbilical cord care and kangaroo mother care [16, 17]. The MOHFW has made a commitment to national scale-up of CNCP guidelines by 2022 [18].

Evaluations of the World Health Organization’s Integrated Management of Childhood Illnesses (IMCI) have revealed that health workers’ newborn assessments with paper forms are frequently incomplete and miss children requiring referral for treatment [19, 20]. Mobile technology, on the other hand, has demonstrated facilitation of exam completion, and adherence to referral and treatment guidelines in LMICs [19]. As part of the United States Agency for International Development’s (USAID) Saving Lives at Birth program, Brigham and Women’s Hospital’s Global Newborn Health Lab partnered with the Johns Hopkins University’s research partnership in Bangladesh (Projahnmo Study Group) and Saving Newborn Lives (Save the Children-Bangladesh) team to design and test a mobile application (app) to aid frontline health workers in the case management of small and sick newborns. The mCNCP (mobile-Comprehensive Newborn Care Package) phone and tablet-based app was designed using concepts of user-centered design to ensure acceptability and ease of navigation. The app features pictures of common newborn conditions from the CNCP training materials and built-in clinical decision support intended to improve identification and guide management and referral of infants needing care. It also includes algorithms to identify and provide special management advice for small and premature infants, based on CNCP and the American Academy of Pediatrics’ Essential Care for Small Babies curriculum [8, 16]. We hypothesized that CHWs using mCNCP would evaluate newborns more completely and efficiently, and would more accurately identify newborn danger signs, identify small and preterm babies, and provide correct management advice, compared to CHWs using standard paper-based forms (pCNCP). Our aims in this study were to test this hypothesis and to assess user satisfaction and preferences to aid future improvements to mCNCP’s development.

## Methods

### The intervention: development and functions of mCNCP

Prior to the development of the app, we conducted key informant interviews with 26 neonatal health stakeholders in Bangladesh, including leaders of non-governmental organizations and professional health societies (*n* = 6) and health workers (*n* = 20), to inform the functionalities of the mCNCP job aid. Participants emphasized that mCNCP should be accessible to CHWs with low technology literacy, include multi-media to increase user understandability, have offline data entry capabilities for field work, and use Android operating systems. We partnered with Saving Newborn Lives to ensure the app integrated the CNCP guidelines and harmonized with their mobile health (m-Health) training materials which they were concurrently developing [14].

**Fig. 1:**
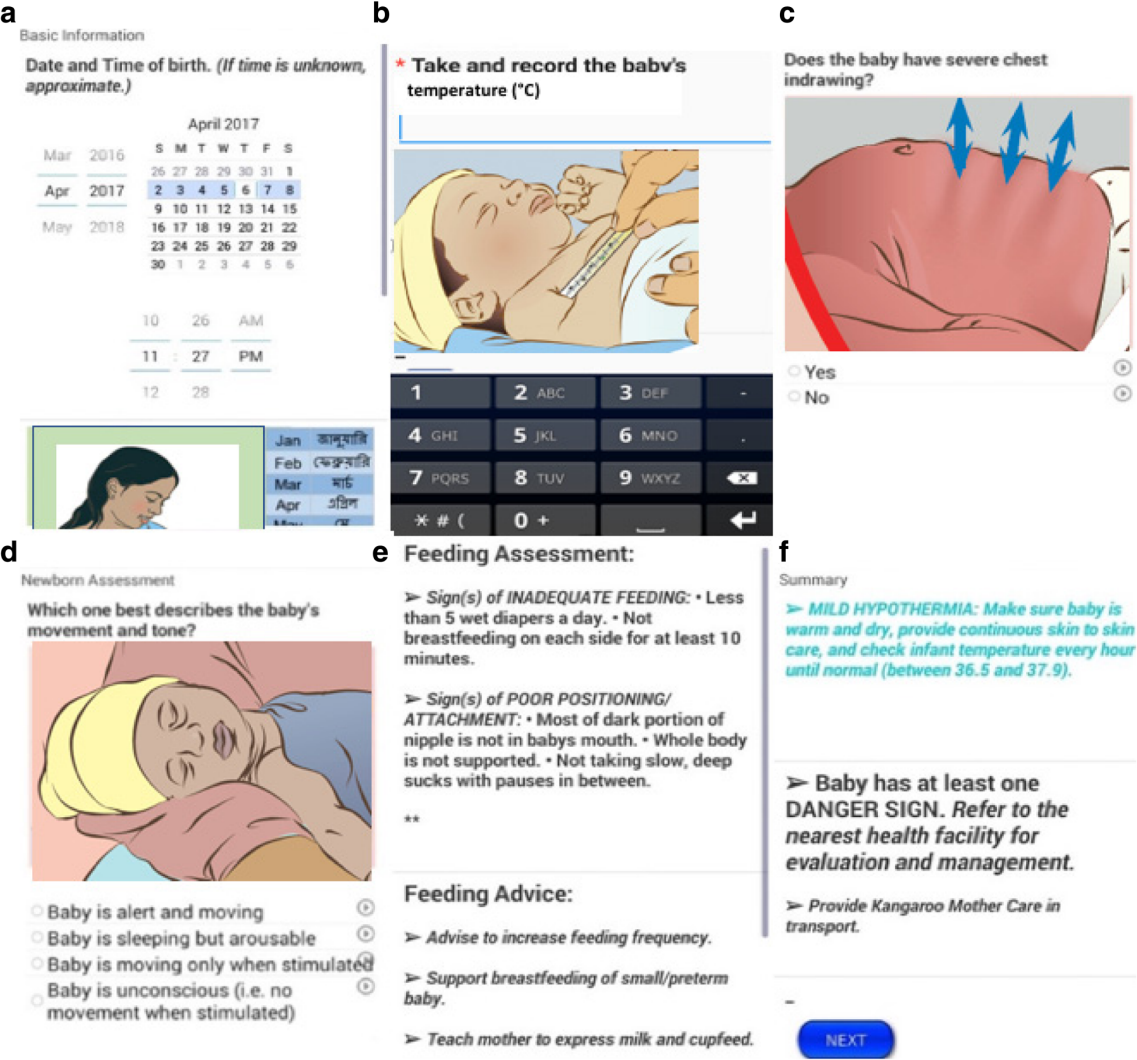
mCNCP Mobile Application Interface (English Translation). Screenshots of the mCNCP mobile application interface (with English-translated instructions), using an Android tablet or phone. Interface images demonstrate prompts for health workers to measure vital signs, assess for conditions and danger signs, and manage conditions. **a** Prompt for health worker to provide date and time of birth using a calendar and drop-down time feature, and a table to translate month names from English to Bangla; **b** Prompt for health worker to measure and record infant’s temperature; **c** Prompt for health worker to assess infant for severe chest indrawing; **d** Prompt for health worker to assess infant’s movement and tone; **e** Generated summary of feeding assessment and feeding advice based on health workers’ responses; **f** Generated summary of infant’s danger signs, conditions, and referral management advice. At the time of the study, mCNCP pages were displayed in the Bangla language. Most images in the mCNCP version used in the study were taken from Bangladesh’s Comprehensive Newborn Care Package (CNCP) guidelines for newborn assessment, that were developed in collaboration with the American Academy of Pediatrics using open source images from Essential Care for Small Babies (ECSB; Provider Guide), Essential Care for Every Baby (ECEB; Parent Guide), and Helping Babies Breathe (HBB; Provider Guide)

mCNCP was developed over the course of six months in an XLS Form, which is converted to Xform standard and HTML by Ona (ona.io), a data-secure platform. The app was designed to guide the health worker through the neonatal assessment, including vital signs, basic newborn examination, and assessment for danger signs. mCNCP has built-in algorithms for classifying small babies (i.e., premature and/or low birth weight), neonatal illnesses (such as sepsis and jaundice), and auto-calculation of weight-loss and gestational age. The app has data entry checks (e.g., answer range constraints, required double entry, and instructions to check responses) and requires questions to be answered before advancing. mCNCP automatically saves responses, generates a summary of findings, provides case management advice and follow-up dates, and uploads all assessments to an online dashboard to display summary charts across infants. Media attachments include visual references from CNCP training materials and a one-minute timer to assist in respiratory rate count. Figure 1 shows examples of the mCNCP’s interface and the summary of findings page generated from a newborn assessment.

Throughout mCNCP’s development, we utilized user-centered design processes, and received iterative feedback from end-users (*n* = 12 CHWs, 5 physicians, and 3 paramedics). The feedback was used to improve its utility, effectiveness, and efficiency. Health workers were consulted in focus groups before the initial coding of mCNCP, and to test two iterations of the app before the study. Research staff, physicians, and paramedics continuously and iteratively tested mCNCP for correctness of referral pathways, calculations, and aesthetic features in the development phase. Bangla translations were reviewed to ensure understandability for the average CHW’s reading level and medical knowledge. User feedback led to the inclusion of calendar (date and time) functions, a one-minute timer for respiratory rate, and summary pages for diagnosis and management recommendations.

The app was accessed on Android tablets through ODKCollect, a free and open-source data collection platform. A paper-based form (pCNCP) was created for the study, with the same assessment questions and order as mCNCP. The same skip logic was in both methods, with pCNCP providing written instructions to the CHW and mCNCP automatically skipping inapplicable assessment sections based on previous responses. Based on the standard format for IMCI assessment, pCNCP had checkboxes next to conditions, and spaces to write case management recommendations, as seen in Additional file 1: Figure S1.

### Training

All CHWs had 3–5 years of previous experience with neonatal care, newborn examination, and IMCI protocols. Twelve CHWs were trained by a study physician with expertise in IMCI and CNCP in three parts (i.e., sessions) over two days. CHWs were first trained in the national CNCP guidelines and provided a booklet with detailed descriptions of the guidelines. The first training session included the CNCP guidelines, identification of small babies (i.e., < 2500 g, < 37 weeks, or < 74 mm foot length), small baby care (i.e., referral thresholds, special advice for thermal care and feeding), and initial introduction to the mCNCP app. Two months later, the second and third training sessions were held in two small groups consisting of 6 CHWs each. The second session was a refresher training on CNCP guidelines, where CHWs received detailed instructions on how to navigate both mCNCP and pCNCP; this included how to respond to questions using either method, how to edit responses, use the calendar function, and properly save completed assessments with mCNCP. On the same day, the third session reviewed small baby care guidelines (including the various referral thresholds based on infant size, age, and danger signs), as well as supervised practice of pCNCP calculations and testing mCNCP on newborns case scenarios. CHWs were given case scenarios with varying combinations of danger signs, mothers’ LMP dates, birth weights and current weights, and temperatures. They were then asked to calculate gestational ages based on LMP and percent weight loss or gain with calculators to practice using pCNCP. CHWs were provided feedback and further instruction when errors occurred on either method during this session. Physicians and paramedics trained in CNCP guidelines acted as gold standard assessors, and were taught how to navigate and save responses on mCNCP. Study recruitment began the afternoons after the third training session.

### Study setting and participant recruitment

Study participants were recruited from two different settings in Sylhet, Bangladesh between March and April 2017. Newborns were eligible if < 28 days old, and not deemed severely ill by their attending physician. The first and primary site of participant recruitment was Sylhet Osmani Medical College Hospital (SOMCH). Ninety-two newborns were enrolled from the pediatric and obstetric wards over a 4-day period. Mothers of recently delivered or admitted newborns were approached consecutively for consent to participate. The second site of participant recruitment was in a community-based setting in the rural Projahnmo research site located in the Zakiganj sub-district (upazilla) in Sylhet district, northeastern Bangladesh. This site was chosen in order to test the feasibility and usability of the app in a community-based setting where CHWs typically assess newborns. Eighteen eligible newborns were consecutively enrolled over a two-week period in the field site.

### Sample size

A sample size of 79 assessments per method was determined to be required to detect a 20 percentage-point difference in the proportion of fully completed neonatal assessment, from 60% in the pCNCP group to 80% in the mCNCP group, with an 80% power and alpha of 5%. This was based on our initial estimate of the proportion of CHWs that would complete assessments, and was supported by previously published data from a similar study in Tanzania [19].

### Data collection

A total of 12 trained female CHWs participated in the study. In SOMCH, for a given study day, 6 CHWs and 3 gold standard assessors (2 physicians and 1 paramedic) conducted assessments on approximately 12 recruited infants each. Randomization occurred at the CHW level, whereby CHWs were randomly assigned CHW identification (ID) numbers, and even or odd ID numbers determined whether their first method of assessment was mCNCP or pCNCP. After 6 newborn assessments, CHWs then alternated to the other method for the remaining 6 neonatal assessments, such that all CHWs used both methods in a particular day. All CHW assessments were compared against a gold standard assessor's assessment by a physician or paramedic using mCNCP, conducted on the same infant during the same shift. Iterative changes were made to mCNCP at the end of each day to improve the smoothness of the mCNCP’s operations but did not affect its content.

### Process indicator measurement and definitions

Efficiency of methods was measured by time in minutes to complete exams, recorded by mCNCP, or calculated from the start and end times written by CHWs for pCNCP. Completeness was defined as CHWs having recorded answers for all conditions, and at least one generated or written care recommendation per assessment. Accuracy of identifying newborn danger signs and correctness of care management advice were determined by comparing each CHWs’ infant assessments against the gold standard assessment for the same infant. Correct classification of danger signs was defined as correctly identifying an infant as having or not having each condition, and correct counseling was defined as providing case management advice when required, as determined by gold standard assessors.

We developed Likert scale questions and surveys for acceptability, functionality, simplicity, and preference for method of assessments to inform further iterations of mCNCP’s design and development (Additional file 2: Figure S2). Survey themes were inspired in part by the Norman Nielson Group’s five components of usability, were administered after the first two days of enrollment, and completed by all 12 CHWs [21]. Focus groups with CHWs and gold standard assessors were conducted at the end of each of the four study days at SOMCH. Discussion themes included interface design, understandability, functionality, future uses, and acceptability of mCNCP by CHWs and the patient community. Key points were summarized from notes and audio recording transcriptions.

### Statistical analysis

Statistical analyses were performed using Stata 14.1 (StataCorp, College Station, Texas) [22]. Average times to complete assessments by each method were compared with two-sided t-tests for differences in means. Proportions were calculated for the completeness of assessments, correct classification of each condition, and correct counseling by each method. The gold standard assessment was used as the reference for correctness. Chi-squared tests were used to compare the significance of differences between proportions. To estimate the odds ratios and 95% confidence intervals of correct classification for each condition comparing mCNCP versus pCNCP, generalized estimating equations (GEE) using a binomial family, logit link, and robust variance were used in order to account for the clustered nature of the data by CHW [23]. Likert scale responses on CHW satisfaction for each method of assessment were compared with two-sided t-tests.

## Results

### CHW characteristics

On average, CHWs were 24.8 years old (± 3.9), had an 11th-grade education (10.8 ± 1.3) and 5 years of CHW work experience (5.1 ± 2.4). While all CHWs reported owning a mobile phone, only 2 (16.7%) reported owning a smart phone and 1 (8.3%) had ever used a tablet.

### Participant study flow

Out of 110 infants enrolled, 9 were excluded from analysis due to problems saving the mCNCP assessment (*n* = 1), missing gold standard physician assessment (*n* = 3), or gold standard assessments missing a CHW assessment (*n* = 5) (Additional file 3: Figure S3). One hundred one unique infants were included in the completeness analysis for most conditions, resulting in 207 assessments at SOMCH (*n* = 83 infants) and in the field (*n* = 18 infants), using either mCNCP (*n* = 103) or pCNCP (*n* = 104). Each CHW completed an average of 11.4 (SD 8.0) assessments included in analysis. Some infants were examined more than once by either of the methods. Assessments from the first day of study enrollment were excluded from correctness analysis as the pCNCP forms had only checkmarks next to each condition following the style of standard IMCI-formatted paper forms. In order to differentiate between an unchecked condition indicating the CHW assessed for the danger sign and determined it a non-issue, as opposed to the CHW did not assess the danger sign and therefore the assessment was incomplete, we added pCNCP response choices of “yes” or “no” for each danger sign. Analysis of correct classification of newborn danger signs by each method of assessment was thus performed on the 170 assessments (*n* = 84 pCNCP; *n* = 86 mCNCP) on 82 unique infants from the study days after this edit to ensure completeness in the paper form was made.

### Efficiency of assessment

mCNCP assessments were completed an average of 6.1 min faster than pCNCP assessments (95% confidence interval, CI: − 8.4, − 3.8; *p* < 0.001). Assessments took an average of 17.5 (± 10.8) and 23.6 (± 11.6) minutes to complete by CHWs using mCNCP and pCNCP, respectively.

### Completeness of neonatal assessment

Neonatal assessments by CHWs were significantly more likely to be fully completed with use of mCNCP (Table 1). Using pCNCP, 23.8% completed the entire assessment for all danger signs (and recorded their findings), compared to 100% of mCNCP CHW assessments (*p* < 0.001). CHWs often neglected to complete or record their findings on pCNCP, or to note whether danger signs were present or absent. They recorded respiratory rates and temperatures, performed calculations of weight-loss and infant postnatal age, and provided specific recommendations for care or referrals less often when using pCNCP. Most notably, mCNCP facilitated gestational age calculations compared to pCNCP (100% vs. 20.7%, *p* < 0.001), assisting identification of preterm infants. The only instance where mCNCP did not markedly improve completeness over the pCNCP was in recording infant weight if available.

**Table 1:** Comparison of Completeness of Neonatal Assessments by CHWs using Paper Forms (pCNCP) and mobile-CNCP (mCNCP)

Completeness of Exams	Paper Form: pCNCP	Mobile App: mCNCP	% Point Difference (mCNCP – pCNCP)	Chi Square Test *P*-Value
Full Assessment Completed^a^	20/84 (23.8%)	86/86 (100%)	76.2	< 0.001
Any Care Management Recommendation Given^b^*	34/104 (32.7%)	103/103 (100%)	67.3	< 0.001
Respiratory Rate Recorded	47/104 (45.2%)	103/103 (100%)	54.8	< 0.001
Temperature Recorded	62/104 (59.6%)	103/103 (100%)	40.4	< 0.001
Weight Recorded^c^	101/104 (97.1%)	102/103 (99.0%)	1.9	0.32
Weight-loss Calculation Completed^d^*	99/104 (95.2%)	103/103 (100%)	4.8	0.024
Gestational Age Calculated (if LMP recorded)*	6/29 (20.7%)	34/34 (100%)	79.3	< 0.001
Postnatal Age Calculated^e^*	77/104 (74.0%)	103/103 (100%)	26.0	< 0.001

^a^All danger signs were assessed, with weight (either birth weight or current weight), temperature, and respiratory rate recorded, and at least one management recommendation given. The version of the IMCI-style paper form on the first day of study enrollment had checkmarks next to newborn conditions, but did not have response choices of “yes” or “no” for each danger sign. This option was included for the remaining days of the study, and thus the first day of assessments was not included in the completeness analysis. ^b^ At least one care recommendation or referral was given. ^c^ Either birth weight or current weight recorded. ^d^ A calculated percent weight loss or gain was recorded (whether correct or incorrect). ^e^Infant’s postnatal age was calculated (whether correct or incorrect).; *mCNCP’s built-in algorithms performed these calculations and generated recommendations

### Participant characteristics

Prevalence of each newborn danger sign and characteristics of participating infants in each method category (pCNCP or mCNCP) are summarized in Table 2, determined by gold standard assessors’ expert classification and CHW classification. The demographics and disease profiles were similar between the comparison and intervention groups by expert classification. Characteristics determined by CHW classifications for each method, however, elucidate when CHWs under- or over-identified exiting danger signs. Using pCNCP, CHWs missed all cases of low birth-weight, and under-estimated small babies and cases of infected umbilicus. Conversely, CHWs with pCNCP over-estimated cases of extremely low weight, poor movement, fever, hypothermia, and breastfeeding issues compared to expert classification. CHWs systematically under-estimated fast-breathing infants by both methods, and over-estimated cases of skin pustules, jaundice, eye infection, and feeding intolerance, by both methods, compared to experts.

**Table 2:** Characteristics of Newborns Observed in Comparison (Paper form: pCNCP) and Intervention Group (Mobile App: mCNCP)

	Expert classification of pCNCP cohort	Expert classification of mCNCP cohort	*P*-Value†	CHW classification of pCNCP cohort	CHW classification of mCNCP cohort	*P*-Value†
Female	26/84 (31.0%)	29/86 (33.7%)	0.70	26/82‡ (31.7%)	32/86 (37.2%)	0.45
Infant post-natal age in days (mean)	6.5 (6.7 SD)	6.5 (6.7 SD)	0.98	6.6 (7.2 SD)	5.6 (6.2 SD)	0.90
Gestational age at birth in weeks (mean)	40.1 (3.2 SD)	40.0 (2.6 SD)	0.89	39.6 (2.2 SD)	39.4 (2.2 SD)	0.86
Low birth-weight^a^*	41/84 (48.8%)	45/86 (52.3%)	0.65	0/84 (0%)	45/86 (52.3%)	< 0.001
Small^b^*	41/84 (48.8%)	45/86 (52.3%)	0.65	9/84 (10.7%)	46/86 (53.5%)	< 0.001
Birth-weight or Current Weight < 1500 g*	6/84 (7.1%)	8/86 (9.3%)	0.61	18/84 (21.4%)	7/86 (8.1%)	0.014
Severe Weight Loss^c^*	21/84 (25.0%)	21/86 (24.4%)	0.93	14/84 (16.7%)	20/86 (23.3%)	0.28
Moderate Weight Loss^d^*	18/84 (21.4%)	18/86 (20.9%)	0.94	22/84 (26.2%)	17/86 (19.8%)	0.32
Poor Movement: Unconscious or moves ONLY when Stimulated	3/84 (3.6%)	3/86 (3.5%)	0.98	34/84 (40.5%)	4/86 (4.7%)	< 0.001
History of Convulsions	19/84 (22.6%)	19/86 (22.1%)	0.93	23/84 (27.4%)	25/86 (29.1%)	0.81
Poor Feeding: Unable to feed OR Stopped feeding well	52/84 (61.9%)	52/86 (60.5%)	0.85	35/84 (41.7%)	47/86 (54.7%)	0.09
Severe Chest In-drawing	3/84 (3.6%)	5/86 (5.8%)	0.49	9/84 (10.7%)	17/86 (19.8%)	0.10
Fast Breathing^e^*	18/84 (21.4%)	15/86 (17.4%)	0.51	9/84 (10.7%)	2/86 (2.3%)	0.026
Umbilicus: red or pus	13/84 (15.5%)	13/86 (15.1%)	0.95	4/84 (4.8%)	18/86 (20.9%)	0.002
Skin Pustules	0/84 (0%)	0/86 (0%)	–	2/84 (2.4%)	6/86 (7.0%)	0.16
Jaundice on Soles or Body	9/84 (10.7%)	10/86 (11.6%)	0.85	18/84 (21.4%)	23/86 (26.7%)	0.42
Eye infection	0/84 (0%)	0/86 (0%)	–	2/84 (2.4%)	5/86 (5.8%)	0.26
Fever^f^*	13/84 (15.5%)	13/86 (15.1%)	0.95	19/84 (22.6%)	10/86 (11.6%)	0.06
Hypothermia ^g^*	5/84 (6.0%)	5/86 (5.8%)	0.97	23/84 (27.4%)	5/86 (5.8%)	< 0.001
Feeding Intolerance^h^	7/84 (8.3%)	6/86 (7.0%)	0.74	22/84 (26.2%)	12/86 (14.0%)	0.046
Problems with breastfeeding^i^	32/84 (38.1%)	32/86 (37.2%)	0.91	62/84 (73.8%)	32/86 (37.2%)	< 0.001

^a^Birth-weight < 2500 g; ^b^Birth-weight < 2500 g or foot- length < 74 mm; ^c^Weight loss > 10% for small infant or > 15% for non-small infant; ^d^Weight loss is 8–10% for small infant or 10–15% for non-small infant; ^e^Respiratory rate > 60 breaths per min; ^f^Temperature > 38 °C or 100.4 °F; ^g^Temperature < 35.5 °C or 95.9 °F; ^h^Infant has at least one condition: chokes, turns blue or pale when feeding, vomits frequently, has distended or tender abdomen, or bloody stools.; ^i^Infant has problems with at least one: waking easily for feeds, breastfeeding for 10+ minutes for per side, sleeping comfortably between feeds, having 5+ wet diapers per day, mother’s breasts haven’t softened.; N: infant participants.; *For mCNCP, these danger signs were identified by the built-in algorithms based on guideline thresholds and CHW assessment responses.; †P-values are from two-sided t-tests for differences in means for infant post-natal age and gestational age at birth, and from chi-squared tests of proportions for all other variables. ‡2 CHWs using pCNCP did not record the infants’ sex.; Of the 170 assessments performed on 82 unique infants, 84 assessments were conducted by CHWs using pCNCP and 86 assessments were conducted by CHWs using mCNCP. Expert classifications were gold standard assessors’ determinations of danger signs (present or not present) using mCNCP. This table shows that the baseline characteristics of infants in the study are similar when comparing proportions of newborn danger signs in the intervention (mCNCP) and comparison (pCNCP) cohorts as determined by the gold standard experts (physicians or paramedics). When comparing the classification of infants in the assessment method cohorts as determined by the CHWs, however, the CHWs tended to over- or under-estimate conditions, and the baseline characteristics thus appear different across the intervention and comparison groups

### Accuracy of neonatal assessment danger sign classifications

Table 3 shows comparisons of correctly classified neonatal conditions, as determined by gold standard assessors, using the mCNCP and pCNCP. Using mCNCP, CHWs were 20.8 times more likely to correctly classify small babies by weight or foot-size (OR: 20.8, (CI: 7.9–48.1), *p* < 0.001), with a 39.4 percentage-point difference from pCNCP classification (*p* < 0.001). CHWs using mCNCP were 13.1 and 13.8 times more likely to correctly classify infants with severe or moderate weight loss (*p* < 0.001), respectively, and significantly more likely to correctly classify danger signs of poor movement, fever, hypothermia, and feeding intolerance. Jaundice was the only condition significantly more likely to be correctly classified by CHWs using pCNCP by chi-squared tests; this may have been due to mCNCP’s use of pictures featuring very yellow, jaundiced babies, which could have anchored CHWs to a higher degree of jaundice than necessary for referral. No significant differences were found for classifying history of convulsions, poor feeding, severe chest in-drawing, fast breathing, infected umbilicus, skin pustules, or eye infection. Identifying feeding intolerance and breast-feeding problems were significantly more likely to be identified by mCNCP only by either chi-squared tests of proportions or odds ratio, but not by both analyses.

**Table 3:** Comparison of Correct Classification of Newborn Conditions by CHWs using Paper Forms (pCNCP) and mobile-CNCP (mCNCP)

Danger Signs	Correctly Classified by pCNCP	Correctly Classified by mCNCP	% Pt Diff (mCNCP - pCNCP)	Chi Square Test *P*-Value	OR	CI for OR	Logit *P*-Value
Low birth-weight^a^*	43/84 (51.2%)	82/86 (95.4%)	44.2	< 0.001	19.5	(7.9, 48.1)	< 0.001
Small^b^*	48/84 (57.1%)	83/86 (96.5%)	39.4	< 0.001	20.8	(7.1, 60.8)	< 0.001
Birth-weight or Current Weight < 1500 g*	70/84 (83.3%)	85/86 (98.8%)	15.5	< 0.001	17.0	(1.8, 160.7)	0.013
Severe Weight Loss^c^*	57/84 (67.9%)	83/86 (96.5%)	28.6	< 0.001	13.1	(4.6, 37.5)	< 0.001
Moderate Weight Loss^d^*	56/84 (66.7%)	83/86 (96.5%)	29.8	< 0.001	13.8	(7.3, 26.1)	< 0.001
Poor Movement: Unconscious or moves ONLY when Stimulated	53/84 (63.1%)	79/86 (91.9%)	28.8	< 0.001	6.6	(2.3, 19.3)	0.001
History of Convulsions	68/84 (81.0%)	70/86 (81.4%)	0.4	0.94	1.0	(0.3, 3.3)	0.96
Poor Feeding: Unable to feed OR Stopped feeding well	59/84 (70.2%)	65/86 (75.6%)	5.4	0.43	1.3	(0.5, 3.2)	0.56
Severe Chest In-drawing	74/84 (88.1%)	68/86 (79.1%)	−9.0	0.11	0.5	(0.2, 1.5)	0.22
Fast Breathing^e^*	71/84 (84.5%)	73/86 (84.9%)	0.4	0.95	1.0	(0.5, 2.0)	0.94
Umbilicus: red or pus	69/84 (82.1%)	61/86 (70.9%)	−11.2	0.09	0.5	(0.2, 1.6)	0.25
Skin Pustules	82/84 (97.6%)	80/86 (93.0%)	−4.6	0.16	0.3	(0.1, 1.5)	0.15
Jaundice on Soles or Body	69/84 (82.1%)	57/86 (66.3%)	−15.8	0.018	0.4	(0.2, 1.1)	0.08
Eye infection	82/84 (97.6%)	81/86 (94.2%)	−3.4	0.26	0.4	(0.1, 2.2)	0.29
Fever^f^*	68/84 (81.0%)	83/86 (96.5%)	15.5	0.001	6.5	(1.3, 31.9)	0.021
Hypothermia ^g^*	62/84 (73.8%)	84/86 (97.7%)	23.9	< 0.001	14.9	(2.7, 82.2)	0.002
Feeding Intolerance^h^	63/84 (75.0%)	74/86 (86.1%)	11.1	0.07	2.1	(1.3, 3.3)	0.003
Problems with breastfeeding^i^	46/84 (54.8%)	62/86 (72.1%)	17.3	0.019	2.1	(0.9, 4.9)	0.07

^a^Birth-weight < 2500 g; ^b^Birth-weight < 2500 g or foot- length < 74 mm; ^c^Weight loss > 10% for small infant or > 15% for non-small infant; ^d^Weight loss is 8–10% for small infant or 10–15% for non-small infant; ^e^Respiratory rate > 60 breaths per min; ^f^Temperature > 38 °C or 100.4 °F; ^g^Temperature < 35.5 °C or 95.9 °F; ^h^Infant has at least one condition: chokes, turns blue or pale when feeding, vomits frequently, has distended or tender abdomen, or bloody stools.; ^i^Infant has problems with at least one: waking easily for feeds, breastfeeding for 10+ minutes for per side, sleeping comfortably between feeds, having 5+ wet diapers per day, mother’s breasts haven’t softened.; *OR* Odds ratio, *CI* Confidence interval; *For mCNCP, these danger signs were identified by the built-in algorithms based on guideline thresholds and CHW assessment responses.; Of the 170 assessments performed on 82 unique infants, 84 assessments were conducted by CHWs using pCNCP and 86 assessments were conducted by CHWs using mCNCP. Expert classifcations were gold standard assessors' determiniations of danger signs (present or not present) using mCNCP. CHWs' assessments were considered correct if their classification of individual danger signs were the same as the gold standard assessment of the newborn's condition

Using mCNCP, CHWs more often provided correct counseling to infants requiring case management, including advising mothers on the need to provide kangaroo mother care, cup-feed, and increase feeding frequency (Additional file 4: Table S1). By chi-squared tests of proportions only, significant differences were found in mCNCP aiding correct counseling for infants requiring referral and special advice for breastfeeding small infants, over pCNCP (91.4% vs 73.8%; *p* = 0.003).

### End-user satisfaction

CHWs significantly preferred mCNCP over pCNCP, and most CHWs thought mCNCP aided ease of understanding instructions and making referral decisions, improved confidence to decide when to refer infants, reduced entry errors, and was the desirable method for future newborn assessments (Additional file 5: Table S2). Results from other sections of the survey can be found in Additional file 6: Tables S3-S5. The majority of CHWs felt confident enough to use mCNCP in the field on their own after the study and believed the app would assist them in newborn assessments and visitation (Additional file 6: Table S3). All CHWs reported feeling more comfortable with and preferred using the app, finding it faster and more accurate in providing recommendations than pCNCP (Additional file 7: Table S4). Additional file 8: Table S5 displays CHWs thoughts on mCNCP’s functionality and understandability, and can be used to inform some of the features of mCNCP for future iterations of the app.

**Fig. 2:**
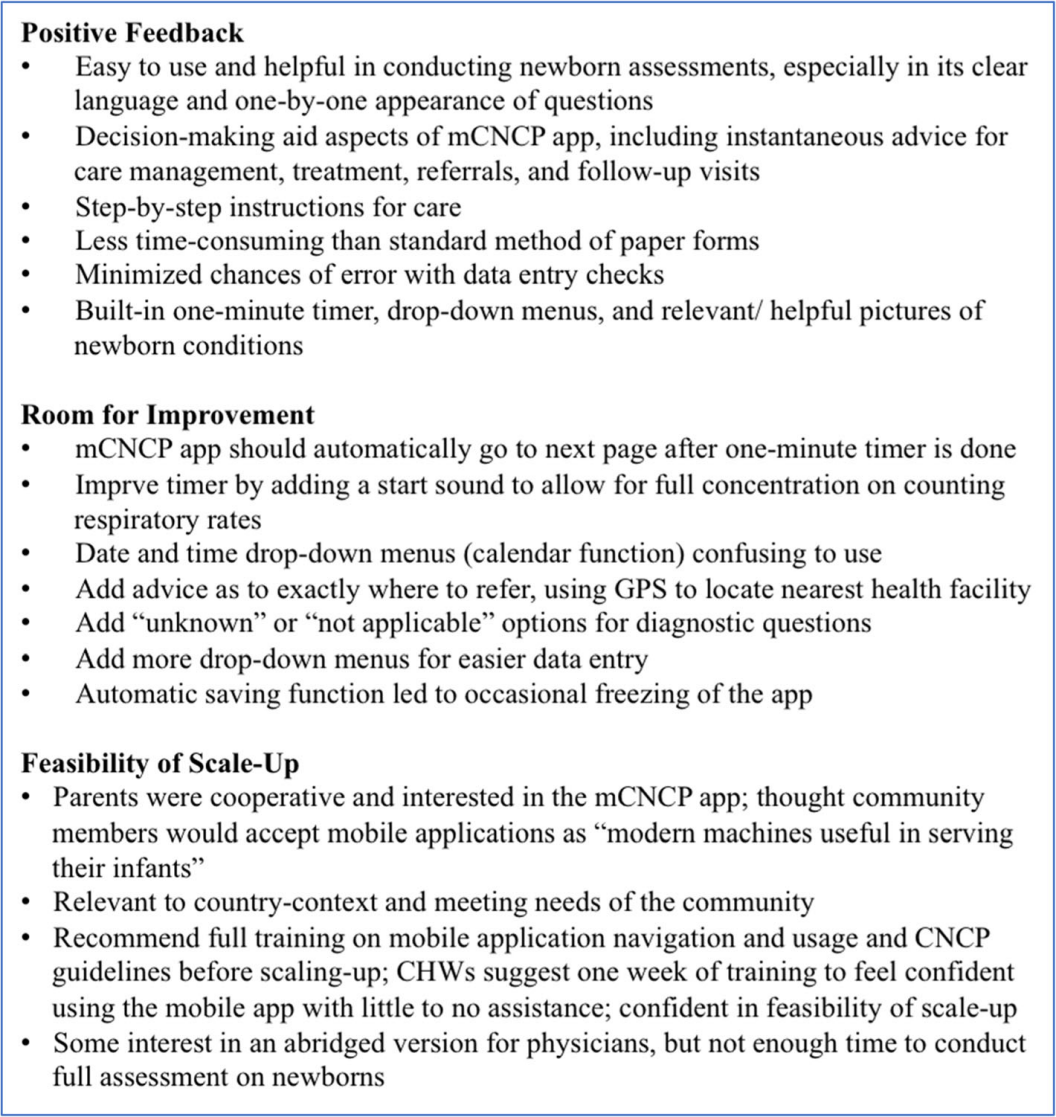
Summary of CHW Focus Group Findings on mCNCP User-Satisfaction and Preferences. Key findings across post-user-testing focus groups with community health workers (CHWs). Topics include positive feedback, identified areas for improvement, and perceptions on the feasibility of mCNCP scale-up

In focus groups, CHWs voiced the desire to use mobile technology in their daily work routine, as they found the built-in calculation tools, one-minute timer, and the generated summary page to be helpful in guiding referrals and counseling. Physicians had informed us that an abbreviated version of the mCNCP would be useful for facility settings in Bangladesh due to the high patient volume and insufficient doctor to patient ratio. To improve the mCNCP’s functionality, CHWs suggested adding a global positioning system (GPS) functionality to aid referral to the nearest facility, considering alternatives for the calendar drop-down function, and discussing with the platform’s developers about the occasional freezing during the automatic saving time-points. A summary of their feedback can be found in Fig. 2.

## Discussion

A mobile application developed in accordance with Bangladeshi newborn care guidelines, mCNCP, aided CHWs to assess and manage common newborn conditions and illnesses. CHWs using mCNCP assessed and managed newborns more efficiently, completely, and correctly compared to those using traditional paper-based forms. Of note, the app aided CHWs in identifying and managing preterm and low birth weight babies, who carry high risks of neonatal morbidity and mortality. CHWs were also more likely to provide appropriate guidance to parents related to feeding and body temperature regulation (e.g., kangaroo mother care). At the end of the study, CHWs using mCNCP reported high levels of satisfaction and confidence in using the technology.

mCNCP aided frontline health workers in a LMIC to more accurately identify neonatal danger signs and risk factors, particularly prematurity and fetal growth restriction (SGA). Prematurity and fetal growth restriction are causes or risk factors for 80% of neonatal deaths [11]. However, these high-risk infants are particularly challenging to identify in LMICs due to limited data, capacity, and resources for antenatal and neonatal care. mCNCP facilitated the auto-calculation of gestational age from mothers’ LMP dates and had built-in algorithms to identify low birth-weight infants based on birth-weight or foot size. CHWs using mCNCP also had enhanced ability to classify conditions of severe or moderate weight loss (by auto-calculating percent weight change), fever or hypothermia (by auto-calculating temperature thresholds), poor movement, and feeding intolerance. The accurate and timely identification of these high-risk conditions and danger signs is the first step required to determine the need for referral and delivery of life-saving interventions, such as respiratory support or antibiotics.

Another strength of mCNCP was its ability to aid health workers in more complex clinical decision-making, by auto-generating referral and management advice based on multiple inputs, thresholds, and calculations. mCNCP’s auto-generation of follow-up visit dates could facilitate timely check-ups to monitor an infant’s health status. Traditional IMCI paper forms use a simple checklist-based approach, and referral is recommended with the presence of any single danger sign [24]. However, for small infants, there are additional considerations and calculations (such as percent weight loss) and more complicated decisions, with lower thresholds for referral [8]. CHWs using mCNCP more often provided correct management advice for small infants requiring increased feeding frequency, cup-feeding, or thermal care, and identified infants requiring referral.

Additionally, m-Health tools may help increase the completion of assessments and reduce data recording errors. mCNCP required CHWs to answer every question before advancing and thus ensured the full completion of the neonatal assessment. The app’s answer constraints, data entry checks, and prompts to review responses may have also contributed to the elimination of errors and improvement upon accurately diagnosing infant danger signs. Similarly, a study in Tanzania reported that an IMCI mobile application significantly improved adherence to guidelines, completeness of assessments, and accuracy of classification of conditions compared to use of traditional paper forms [19]. Studies in China comparing smartphone applications to pen-and-paper forms had similar results in reducing data recording errors [25], and finding the paper method to be more error-prone [26].

CHWs preferred using mCNCP over pCNCP, stating it was understandable, functional, easy to use, and faster to complete, and voiced a desire to use mobile applications in their daily work. Given their previous inexperience with smart phones or tablets, this feedback is encouraging for the acceptance, capabilities, and potential health implications of future m-Health interventions and indicates low-level health workers are capable of learning the technology quickly. As the devices become increasingly affordable, countries can consider the potential for scalability of m-Health solutions operated by CHWs.

mCNCP is uniquely situated as a neonatal data collection tool for frontline health workers, created with user-centered design and iterative processes. Many m-Health interventions involve text message correspondence between health care providers and pregnant women or mothers to increase maternal and newborn service utilization, including two in Bangladesh: MAMA and JiVitA [27]. To our knowledge, at the time we did not identify other m-Health solutions in Bangladesh that provide decision-making support to maternal or newborn health providers [26, 28]. Through user-centered design, we have addressed some of the challenges past m-Health interventions have faced, including providing detailed care advice and addressing low literacy levels of our users, including technology and medical literacy [26]. Incorporating features such as GPS, video and audio instructions, and the ability to take and store pictures of patients, per stakeholders’ suggestions, could be beneficial for tracking patient outcomes and improving diagnoses [26]. A Bangladesh study found that mobile applications with a feature to allow for real-time access to patient medical data led to more timely triage and initial treatment of patients, supporting statements from our key stakeholders [26]. Integration of mCNCP with the existing health management information system in Bangladesh may be beneficial to the MOHFW and health administrators to monitor and track the epidemiology of neonatal diseases.

### Limitations

In this evaluation, we did not assess the impact of the mCNCP on provider to parent communication, care-seeking behaviors, or health outcomes. Previous studies have demonstrated the ability of m-Health tools to improve communication of diagnoses between providers and patients, and parents’ recall of case management advice [29]. Mothers’ health-seeking behaviors and healthcare utilization throughout pregnancy, delivery, and the newborn period have also improved with the use of m-Health systems and devices [26, 27, 30]. Studying the impact of mCNCP on patient behavior would further the understanding of its implications on improving patient health. We experienced some limitations in developing mCNCP for our intended end-users, and pose lessons learned that could benefit future m-Health tools and programs. Ona, mCNCP’s platform, while easy to operate and program, had limits to functionality and appearance at the time of the app’s development. Some CHWs found mCNCP’s drop-down calendar function difficult to use, and we were unable to use the luni-solar Bengali calendar with this feature. As previously mentioned, the initial pCNCP paper forms in our study were based on the standard IMCI format, which did not allow for accurate assessment of whether the person completing the form assessed for all danger signs or skipped the section. This issue was rectified for our paper forms part-way through our study. We experienced some data loss at the beginning of the study, losing 2 mCNCP assessments from the first day of patient enrollment at SOMCH and 1 from the first day of field-based assessments, indicating a brief user learning period is needed to ensure all mCNCP forms are saved properly. Furthermore, for future iterations of mCNCP, data needs to be fed back into Health Management Information Systems for monitoring purposes, which we did not have the opportunity to develop for this study.

## Conclusions

This study demonstrates that CHWs with little to no experience using mobile tablets can use and operate a mobile application for newborn assessments after a two-day training with user-testing and supervision. mCNCP serves as a decision-aid tool, which has shown to improve completeness and efficiency of newborn assessments, classification of illness, and adherence to clinical guidelines compared to the standard method of paper forms. User-centered design ensured the usability, functionality, and acceptability of the mobile application by CHWs, who voiced their satisfaction with and preference for mCNCP as a method of assessment. The impact of m-Health tools such as mCNCP on health outcomes should be rigorously evaluated. Furthermore, future efforts should be made to develop systems to integrate the results and findings of such tools into Health Information Management Systems in order to guide regional and national level monitoring and planning.

## Additional files

Additional file 1: **Figure S1.** **pCNCP Paper Form for Newborn Assessments (English Translation).** Paper form (pCNCP) developed for the current study to compare against community health workers’ (CHWs') performance with the mCNCP mobile application. (DOCX 18 kb)

Additional file 2: **Figure S2.** **Likert Scale and Survey Questions to Compare mCNCP and pCNCP Preferences, Functionality, Usability, and Acceptability.** Survey questionnaire developed to compare community health workers’ (CHWs') preferences and perceptions of mCNCP mobile application and pCNCP paper form in order to qualitatively compare newborn assessment methods. (DOCX 21 kb)

Additional file 3: **Figure S3.** **Participant Study Flowchart.** Flowchart of participants' study involvement from enrollment to analysis, including loss to follow-up and analysis exclusions. (DOCX 73 kb)

Additional file 4: **Table S1.** **Comparison of Newborn Management Counseling by CHWs using Paper Forms (pCNCP) and mobile-CNCP (mCNCP).** Results comparing correct referral and management advice by community health workers (CHWs) performing newborn assessments with pCNCP and mCNCP, with a physician assessment as the gold standard for correct newborn management counseling. Proportions of correctly identified referral and recommendation advice for each method of assessment. Percentage-point differences, chi-squared tests of differences between proportions, and odds ratios comparing mCNCP and pCNCP assessments. (DOCX 13 kb)

Additional file 5: **Table S2.** **Comparison of CHW Likert Scale Responses for Usability, Functionality, and Acceptability of mCNCP and pCNCP.** Results comparing community health workers’ (CHWs') experiences with mCNCP mobile application and pCNCP paper form, including the ease of use, level of confidence, and desire to use the method for future assessments. (DOCX 13 kb)

Additional file 6: **Table S3.** **CHW Likert Scale Responses about mCNCP Practicality and Preferences.** Results summarizing community health workers’ (CHWs') experiences with mCNCP, including level of confidence to use on their own in the field, perceptions of the utility of the mCNCP mobile application to assist in identifying newborn danger signs and providing advice, ease of use, and understandability. (DOCX 34 kb)

Additional file 7: **Table S4.** **CHW Binary Survey Responses Comparing Preferences and Functions of mCNCP and pCNCP.** Results comparing community health workers’ (CHWs') experiences with mCNCP mobile application and pCNCP paper form, wherein CHWs selected which method of assessment they found faster, preferred, felt more comfortable using, thought to be more accurate in providing referrals and management advice, and which method led to more mistakes. (DOCX 41 kb)

Additional file 8: **Table S5.** **CHW Survey Responses about mCNCP Interface and Features.** Results summarizing community health workers’ (CHWs') opinions of the simplicity, functionality, and usability of mCNCP’s interface and features. (DOCX 16 kb)

## Data Availability

Datasets generated during the current study are available from the corresponding author upon reasonable request.

## Abbreviations

App(s): Application(s)
CHW: Community health worker
CNCP: Comprehensive Newborn Care Package
GPS: Global positioning system
ID: Identification
IMCI: Integrated Management of Childhood Illnesses
LMIC: Low- or middle-income country
LMP: Last menstrual period
mCNCP: mobile Comprehensive Newborn Care Package
MOHFW: Ministry of Health and Family Welfare
pCNCP: standard paper-based form comparable to mCNCP
SGA: Small-for-gestational age
SOMCH: Sylhet Osmani Medical College Hospital
