# Supplementary material for: Development and evaluation of a mobile application for case management of small and sick newborns in Bangladesh
Source: BMC Med Inform Decis Mak. 2019 Jun 20;19:116. doi: 10.1186/s12911-019-0835-7 (PMC6585142; doi:10.1186/s12911-019-0835-7)
Supplement: Supplementary file 1 — Figure S1. pCNCP Paper Form for Newborn Assessments (English Translation). Paper form (pCNCP) developed for the current study to compare against community health workers’ (CHWs') performance with the mCNCP mobile application. (DOCX 18 kb) [file 12911_2019_835_MOESM1_ESM.docx]

*Where are you conducting the exam? (Circle* ***Osmani*** *or* ***Field****) CHW Study ID: ________________ Baby Study ID: _______________*

*Today’s Date: _________________ Start Time of Assessment: ______________*

*CHW’s Name: __________________ CHW’s Gender: ______________________*

***Address and Contact Information (for Field Visits only):***

*Mother’s Name: ____________________ Father’s Name: __________________ Baby’s Name: ______________*

*District: ___________Upazilla: _________ Union: ________ Village: _________ House: ________*

*Parent’s or Neighbor’s Mobile Phone Number: _______________*

***Basic Information:***

*Baby’s Sex: ____* Baby date of birth: ___________________ Time of birth: ________ Baby’s Age:_____ Days

Day Month Year

First day of last menstrual period: (if unknown, indicate “unknown”) __________ Gestational age of baby at birth: ________ weeks _____ days

Baby’s birthweight (if unknown, indicate “unknown”) __________________ grams

Baby’s current weight (if scale not available, indicate “unknown”) ____________grams

If neither weight is available, please measure foot length _______________mm.

***Calculate the baby’s weight loss or gain:***

***Weight Loss= (birth weight - current weight)/ birth weight * 100***

Baby’s weight loss is _______%

| **Danger Signs** | **Provider Recommendation** |
| --- | --- |
| - **Unconscious/ No movement at all/ drowsy**   ☐ Yes ☐ No ☐ Don’t Know   - **Less movement than normal/ movement only when stimulated**   ☐ Yes ☐ No ☐ Don’t Know   - **Convulsion/History of convulsion**   ☐ Yes ☐ No ☐ Don’t Know   - **Unable to feed**   ☐ Yes ☐ No ☐ Don’t Know   - **Weight <1500 grams**   ☐ Yes ☐ No ☐ Don’t Know   - **Severe chest in-drawing**   ☐ Yes ☐ No ☐ Don’t Know   - **Fast Breathing (60 or more per minute)**   ☐ Yes ☐ No ☐ Don’t Know   - **Umbilical redness or draining pus from umbilicus**   ☐ Yes ☐ No ☐ Don’t Know   - **Skin pustules**   ☐ Yes ☐ No ☐ Don’t Know   - **Eye infection**   ☐ Yes ☐ No ☐ Don’t Know   - **Raised Temperature/ Fever (>100.4 °F/38.0 °C)**   ☐ Yes ☐ No ☐ Don’t Know |  |
| - **Hypothermia (<95.9 °F/ 35.5 °C)**   ☐ Yes ☐ No ☐ Don’t Know |  |
| - **Not feeding well**   ☐ Yes ☐ No ☐ Don’t Know |  |
| - **Severe Jaundice: jaundice detected on soles**   ☐ Yes ☐ No ☐ Don’t Know   - **Severe Jaundice: jaundice elsewhere (i.e. forehead, abdomen, thighs)**   ☐ Yes ☐ No ☐ Don’t Know |  |
| - **Severe weight loss**   ☐ Yes ☐ No ☐ Don’t Know |  |

*If baby is* ***small or preterm****:*

| evaluate the baby for **feeding intolerance**. Ask the mother the following questions. Does the baby: | **Provider Recommendation** |
| --- | --- |
| - **choke, turn blue or pale when feeding?**   ☐ Yes ☐ No ☐ Don’t Know   - **vomit frequently?**   ☐ Yes ☐ No ☐ Don’t Know   - **have distended or tender abdomen?**   ☐ Yes ☐ No ☐ Don’t Know   - **have bloody stools?**   ☐ Yes ☐ No ☐ Don’t Know |  |

| **Other** | **Provider Recommendation** |
| --- | --- |
| - **Significant weight loss**   ☐ Yes ☐ No ☐ Don’t Know |  |
| - **Mild Hypothermia (95.9-97.6 °F/ 35.5-36.4 °C)**   ☐ Yes ☐ No ☐ Don’t Know |  |

**Feeding Questions**

| Evaluate the baby’s effectiveness at breastfeeding and whether the baby is adequately fed. Ask the mother the following questions. Does the baby: | **Provider Recommendation** |
| --- | --- |
| - **wake easily for feeds and show feeding readiness cues?**   ☐ Yes ☐ No ☐ Don’t Know   - **breastfeed for at least 10 minutes per side?**   ☐ Yes ☐ No ☐ Don’t Know   - **sleep comfortably between feedings every 2-3 hours?**   ☐ Yes ☐ No ☐ Don’t Know   - **have 5 or more wet diapers per day?**   ☐ Yes ☐ No ☐ Don’t Know   - **(if baby is 7 days or older) Do mother’s breasts soften after feeding?**   ☐ Yes ☐ No ☐ Don’t Know |  |

**Breastfeeding Assessment**

Are you able to observe the mother breastfeeding at this time?

- No, skip to the final section on the next page.
- Yes, answer the following questions. *Ask the mother to start breastfeeding and observe.*

| Assess for signs of **good attachment:** | **Provider Recommendation** |
| --- | --- |
| - **Baby’s mouth is wide open.**   ☐ Yes ☐ No ☐ Don’t Know   - **Most of the dark portion of the nipple (areola) is in the baby’s mouth.**   ☐ Yes ☐ No ☐ Don’t Know   - **Lower lip is turned outwards.**   ☐ Yes ☐ No ☐ Don’t Know   - **Chin is touching breast.**   ☐ Yes ☐ No ☐ Don’t Know |  |
| Now assess the signs of **good positioning.** Is the baby’s: |  |
| - **neck straight (not flexed)?**   ☐ Yes ☐ No ☐ Don’t Know   - **whole body supported?**   ☐ Yes ☐ No ☐ Don’t Know   - **head and body in a straight line?**   ☐ Yes ☐ No ☐ Don’t Know |  |
| Now assess the signs of **effective feeding:** |  |
| - **Is the baby taking slow, deep sucks with pauses in between?**   ☐ Yes ☐ No ☐ Don’t Know   - **Is the baby’s swallowing visible?**   ☐ Yes ☐ No ☐ Don’t Know |  |

Did attachment and positioning improve after counseling the mother?

- There was no need to counsel the mother.
- Yes.
- No, attachment/ positioning did not improve but the baby is still able to breastfeed. *(Continue to provide breastfeeding support to improve position and attachment.)*
- No, baby is unable to breastfeed after several attempts. *(Advise mother to express milk and cup feed at the end of the exam.)*

***What Conditions did you diagnose the baby with?***

|  |
| --- |

***What Advice did you give the family? (e.g. referral, thermal care, feeding advice, etc)***

|  |
| --- |

**Referrals**

Did you refer the baby for danger signs?

- No
- Yes

Did the family comply with the referral?

- No referral was necessary.
- No
- Yes

*End Time of Assessment: ______________*
