# Supplementary material for: Development and evaluation of a mobile application for case management of small and sick newborns in Bangladesh
Source: BMC Med Inform Decis Mak. 2019 Jun 20;19:116. doi: 10.1186/s12911-019-0835-7 (PMC6585142; doi:10.1186/s12911-019-0835-7)
Supplement: Supplementary file 2 — Figure S2. Likert Scale and Survey Questions to Compare mCNCP and pCNCP Preferences, Functionality, Usability, and Acceptability. Survey questionnaire developed to compare community health workers’ (CHWs') preferences and perceptions of mCNCP mobile application and pCNCP paper form in order to qualitatively compare newborn assessment methods. (DOCX 21 kb) [file 12911_2019_835_MOESM2_ESM.docx]

**(Usability/ Ease of Use)**

***1a) “Learnability” (Ability to Learn the App):*** (These questions are about how easy it is to learn how to use and operate the app,and compares your ability to learn and use the app to the paper form. Please check the answer that best describes how much you agree to each statement about the mobile app:)

| Statement: | Completely Disagree | Mostly Disagree | Slightly Disagree | Slightly Agree | Mostly Agree | Completely Agree |
| --- | --- | --- | --- | --- | --- | --- |
| I feel confident to use the mobile app in the field on my own. |  |  |  |  |  |  |
| I feel confident to use the paper form in the field on my own. |  |  |  |  |  |  |
| It is easy to use the mobile app after using it a few times. |  |  |  |  |  |  |
| It is easy to use the paper form after using it a few times. |  |  |  |  |  |  |
| The mobile app will help CHWs assess newborns. |  |  |  |  |  |  |
| The app will help CHWs identify danger signs. |  |  |  |  |  |  |
| The app will help CHWs refer the baby when necessary. |  |  |  |  |  |  |
| The app will help CHWs give the family feeding advice. |  |  |  |  |  |  |
| The app will help CHWs schedule follow-up with the baby. |  |  |  |  |  |  |

***1b)*** How long do you think it would take CHWs to learn how to use the mobile app and feel confident using it in the field?

|  |
| --- |

***2b) Errors***

|  | Never | Very Rarely | Rarely | Occasionally | Frequently | Very Frequently |
| --- | --- | --- | --- | --- | --- | --- |
| How often did you feel you made errors when using the mobile app? |  |  |  |  |  |  |
| How often did you feel you made errors when using the paper form? |  |  |  |  |  |  |

***2b)*** What were the errors when using the mobile app?

|  |
| --- |

***2c)*** Were you able to correct any errors you made during the newborn assessment using the mobile app?

- Yes
- No

|  | Not easy | Slightly Easy | Moderately Easy | Easy | Very Easy |
| --- | --- | --- | --- | --- | --- |
| If “yes”, how easy was it to correct these errors on the app? |  |  |  |  |  |

***2d)*** What were the errors when using the paper form?

|  |
| --- |

***2e)*** Were you able to correct any errors you made during the newborn assessment using the paper form?

- Yes
- No

|  | Not easy | Slightly Easy | Moderately Easy | Easy | Very Easy |
| --- | --- | --- | --- | --- | --- |
| If “yes”, how easy was it to correct errors in the paper form? |  |  |  |  |  |

***2f)*** Which method did you find to be faster?

*Circle* ***Mobile App*** *or* ***Paper Form***

***2g)*** Which method do you feel led to the most mistakes?

*Circle* ***Mobile App*** *or* ***Paper Form***

***2h)*** Which method did you feel more comfortable using?

*Circle* ***Mobile App*** *or* ***Paper Form***

| Why? |
| --- |

***2i)*** Which method do you think was more accurate in recommending referrals and providing advice?

*Circle* ***Mobile App*** *or* ***Paper Form***

| Why? |
| --- |

***2j)*** Which method would you prefer to use?

Circle **Mobile App** or **Paper Form**

| Why? |
| --- |

**(Content/ Language, Decision-Making, Satisfaction)**

***3a)*** These questions are about how easy the app was to understand and use, and your feelings about the app’s decision-making. Please check the answer that best describes how much you agree to each statement about the mobile app:

| Statement: | Completely Disagree | Mostly Disagree | Slightly Disagree | Slightly Agree | Mostly Agree | Completely Agree |
| --- | --- | --- | --- | --- | --- | --- |
| The questions on the app are easy to understand. |  |  |  |  |  |  |
| It is easy to correctly follow instructions on the mobile app. |  |  |  |  |  |  |
| It is easy to correctly follow instructions on the paper form |  |  |  |  |  |  |
| It is easy to make referral decisions using the app. |  |  |  |  |  |  |
| It is easy to make referral decisions using the paper form |  |  |  |  |  |  |
| It is satisfying to use the app for newborn assessments. |  |  |  |  |  |  |
| It is satisfying to use the paper form for newborn assessments. |  |  |  |  |  |  |
| I feel confident that I can decide on when to refer babies when using the app. |  |  |  |  |  |  |
| I feel confident that I can decide on when to refer babies when using the paper form. |  |  |  |  |  |  |
| I want to use the mobile app for future newborn assessments. |  |  |  |  |  |  |
| I want to use the paper form for future newborn assessments. |  |  |  |  |  |  |

***4a) Language***

***4ai)*** Are the questions easy to understand in Bangla?

- Yes
- No

***4aii)*** Should the language of the questions be more formal (i.e. use of medical terms) or do you prefer simplified translations?

- Formal language (i.e. medical terms)
- Simplified translations

***4aiii)*** If there were any phrases that were difficult to understand, please list the Bangla words/ phrases below:

|  |
| --- |

***5a) Simplicity/ Understandability***

***5ai)*** Is the Bangla font legible on the app?

- Yes
- No

***5b)*** Are you able to read the captions on the pictures? (Check “yes” or “no” next to the description of the pictures

| - Yes - No | Male and Female pictures |
| --- | --- |
| - Yes - No | Other: |

***5c)*** Did you find the pictures and diagrams in the app clear and easy to use?

- Yes
- No

If not, which ones did you not understand? (i.e. caution sign- yellow triangle):

|  |
| --- |

***5d)*** Are you able to press **all** of the buttons on the app with ease?

- Yes
- No

If “no”, which buttons were difficult to press?

|  |
| --- |

***5e)*** Did you experience any problems when navigating the app? (check one answer next to each)

| - Yes - No | Swiping to move to the next page (or screen) of the app |
| --- | --- |
| - Yes - No | Scrolling to view the whole summary page (or other pages) |
| - Swiping - Next Button | Do you prefer the “swiping” function to move to the next page, or would you prefer a “Next” button to move to the next page? |
| - Yes - No | Was it easy for you to “swipe” back to previous pages to make corrections? |
