# Supplementary figures and images for: Development and evaluation of a mobile application for case management of small and sick newborns in Bangladesh
Source: BMC Med Inform Decis Mak. 2019 Jun 20;19:116. doi: 10.1186/s12911-019-0835-7 (PMC6585142; doi:10.1186/s12911-019-0835-7)

**
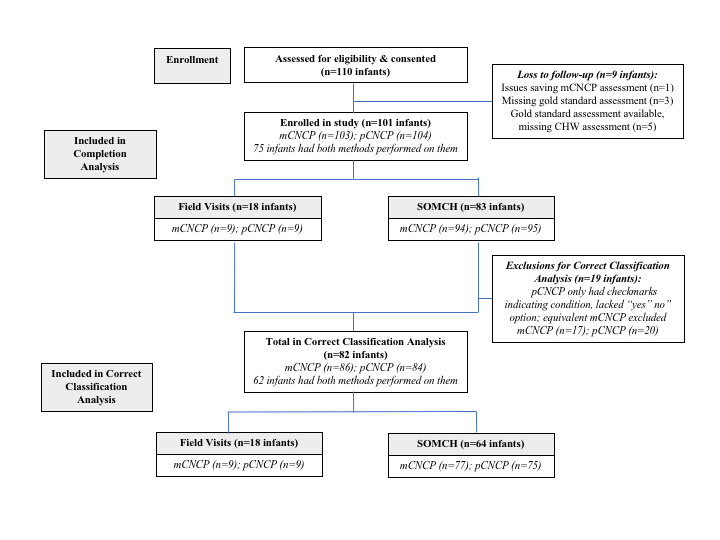
**

Supplement: Supplementary file 3 — Figure S3. Participant Study Flowchart. Flowchart of participants' study involvement from enrollment to analysis, including loss to follow-up and analysis exclusions. (DOCX 73 kb) [file 12911_2019_835_MOESM3_ESM.docx]
