# Supplementary material for: Development and evaluation of a mobile application for case management of small and sick newborns in Bangladesh
Source: BMC Med Inform Decis Mak. 2019 Jun 20;19:116. doi: 10.1186/s12911-019-0835-7 (PMC6585142; doi:10.1186/s12911-019-0835-7)
Supplement: Supplementary file 4 — Table S1. Comparison of Newborn Management Counseling by CHWs using Paper Forms (pCNCP) and mobile-CNCP (mCNCP). Results comparing correct referral and management advice by community health workers (CHWs) performing newborn assessments with pCNCP and mCNCP, with a physician assessment as the gold standard for correct newborn management counseling. Proportions of correctly identified referral and recommendation advice for each method of assessment. Percentage-point differences, chi-squared tests of differences between proportions, and odds ratios comparing mCNCP and pCNCP assessments. (DOCX 13 kb) [file 12911_2019_835_MOESM4_ESM.docx]

| **Advice Correctly Given to Infants Requiring Counseling** | **Paper Form: pCNCP** | **Mobile App: mCNCP** | **% Pt Diff (mCNCP - pCNCP)** | **Chi Square Test**  **P-Value** | **OR** | **CI for OR** | **Logit P-Value** |
| --- | --- | --- | --- | --- | --- | --- | --- |
| Refer to hospital | 62/84 (73.8%) | 74/81 (91.4%) | 17.6 | 0.003 | 3.8 | (1.0, 14.0) | 0.050 |
| Wrap baby in warm clothing | 3/33 (9.1%) | 28/32 (87.5%) | 78.4 | <0.001 | 70.0 | (9.6, 511.0) | <0.001 |
| Increased feeding frequency | 4/30 (13.3%) | 13/31 (41.9%) | 28.6 | 0.013 | 4.7 | (1.6, 14.0) | 0.006 |
| Provide breastfeeding guidance | 1/3 (33.3%) | 0/3 (0%) | -33.3 | 0.27 | - | - | - |
| ***Recommendations for Small Infants:*** | | | | | | | |
| Express milk and cup-feed | 7/56 (12.5%) | 48/54 (88.9%) | 76.4 | <0.001 | 56.0 | (15.2, 205.6) | <0.001 |
| Provide Kangaroo Mother Care | 5/47 (10.6%) | 45/47 (95.7%) | 85.1 | <0.001 | 189.0 | (23.1, 1546.8) | <0.001 |
| Provide special advice on breastfeeding small babies | 0/51 (0%) | 50/53 (94.3%) | 94.3 | <0.001 | - | - | - |
| *Denominators for pCNCP and mCNCP were infants requiring counseling as determined by gold standard assessments.;*  *OR: odds ratio; CI: confidence interval; For mCNCP, all management and referral advice were automatically generated by the app’s built-in algorithms based on the CHW assessment responses.* | | | | | | | |
