# Supplementary material for: Development and evaluation of a mobile application for case management of small and sick newborns in Bangladesh
Source: BMC Med Inform Decis Mak. 2019 Jun 20;19:116. doi: 10.1186/s12911-019-0835-7 (PMC6585142; doi:10.1186/s12911-019-0835-7)
Supplement: Supplementary file 5 — Table S2. Comparison of CHW Likert Scale Responses for Usability, Functionality, and Acceptability of mCNCP and pCNCP. Results comparing community health workers’ (CHWs') experiences with mCNCP mobile application and pCNCP paper form, including the ease of use, level of confidence, and desire to use the method for future assessments. (DOCX 13 kb) [file 12911_2019_835_MOESM5_ESM.docx]

| **Theme** | **Statement** | **Paper Form:**  **pCNCP** | | | **Mobile App: mCNCP** | | | **Comparison** |
| --- | --- | --- | --- | --- | --- | --- | --- | --- |
|  |  | **Mean** | **SD** | **N** | **Mean** | **SD** | **N** | **T-test P-Value of Difference** |
| Usability^a^ | It is easy to correctly follow instructions. | 3.3 | 0.5 | 12 | 5 | 0 | 12 | <0.001 |
| Functionality^a^ | It is easy to make referral decisions. | 3.3 | 0.6 | 12 | 4.4 | 0.5 | 12 | <0.001 |
| Functionality^a^ | I feel confident that I can decide on when to refer babies. | 3.2 | 0.7 | 12 | 4.7 | 0.8 | 12 | <0.001 |
| Functionality^b^ | How often did you feel you made errors? | 2.3 | 0.5 | 12 | 2.8 | 1.2 | 12 | 0.20 |
| Acceptability^a^ | I want to use this method for future newborn assessments. | 2.8 | 1.0 | 12 | 4.6 | 0.5 | 11 | <0.001 |
| *^a^Answer choices were: 0 (Completely Disagree), 1 (Mostly Disagree), 2 (Slightly Disagree), 3 (Slightly Agree), 4 (Mostly Agree), 5 (Completely Agree); ^b^Answer choices were: 0 (Very Frequently), 1 (Frequently), 2 (Occasionally), 3 (Rarely), 4 (Very Rarely), 5 (Never); SD: Standard Deviation; N: number of CHWs who answered* | | | | | | | | |
