# Supplementary material for: Development and evaluation of a mobile application for case management of small and sick newborns in Bangladesh
Source: BMC Med Inform Decis Mak. 2019 Jun 20;19:116. doi: 10.1186/s12911-019-0835-7 (PMC6585142; doi:10.1186/s12911-019-0835-7)
Supplement: Supplementary file 6 — Table S3. CHW Likert Scale Responses about mCNCP Practicality and Preferences. Results summarizing community health workers’ (CHWs') experiences with mCNCP, including level of confidence to use on their own in the field, perceptions of the utility of the mCNCP mobile application to assist in identifying newborn danger signs and providing advice, ease of use, and understandability. (DOCX 34 kb) [file 12911_2019_835_MOESM6_ESM.docx]

| **Theme** | **Statement** | **Mean** | **SD** | **N** |
| --- | --- | --- | --- | --- |
| Acceptability^a^ | I feel confident to use the app in the field on my own. | 4.9 | 0.3 | 12 |
| Functionality^a^ | The mobile app will help CHWs assess newborns. | 5.0 | 0.0 | 12 |
| Functionality^a^ | The app will help CHWs identify danger signs. | 4.6 | 0.8 | 12 |
| Functionality^a^ | The app will help CHWs refer the baby when necessary. | 4.2 | 0.8 | 12 |
| Functionality^a^ | The app will help CHWs give the family feeding advice. | 4.4 | 0.9 | 12 |
| Functionality^a^ | The app will help CHWs schedule follow-up with the baby. | 4.5 | 0.5 | 12 |
| Functionality^b^ | How easy was it to correct these errors on the app? | 3.6 | 0.5 | 12 |
| Usability^a^ | The questions on the app are easy to understand. | 4.9 | 0.3 | 12 |
| Usability^a^ | It is easy to use the mobile app after using it a few times. | 4.6 | 0.5 | 12 |
| *^a^Answer choices were: 0 (Completely Disagree), 1 (Mostly Disagree), 2 (Some-what Disagree), 3 (Some-what Agree), 4 (Mostly Agree), 5 (Completely Agree); ^b^Answer choices were: 0 (Very Difficult), 1(Difficult), 2 (Neutral), 3(Easy), 4(Very Easy); SD: Standard Deviation; N: number of CHWs who answered* | | | | |
