# Supplementary material for: Development and evaluation of a mobile application for case management of small and sick newborns in Bangladesh
Source: BMC Med Inform Decis Mak. 2019 Jun 20;19:116. doi: 10.1186/s12911-019-0835-7 (PMC6585142; doi:10.1186/s12911-019-0835-7)
Supplement: Supplementary file 7 — Table S4. CHW Binary Survey Responses Comparing Preferences and Functions of mCNCP and pCNCP. Results comparing community health workers’ (CHWs') experiences with mCNCP mobile application and pCNCP paper form, wherein CHWs selected which method of assessment they found faster, preferred, felt more comfortable using, thought to be more accurate in providing referrals and management advice, and which method led to more mistakes. (DOCX 41 kb) [file 12911_2019_835_MOESM7_ESM.docx]

|  | | | | | |
| --- | --- | --- | --- | --- | --- |
| **Theme** | **Question** | **Paper Form: pCNCP** | **Mobile App: mCNCP** | **N** |  |
| Simplicity | Which method did you find to be faster? | 0% (0/0) | 100% (9/9) | 9 |  |
| Acceptability | Which method would you prefer to use? | 0% (0/0) | 100% (8/8) | 8 |  |
| Usability | Which method did you feel more comfortable using? | 0% (0/0) | 100% (9/9) | 9 |  |
| Functionality | Which method do you think was more accurate in recommending referrals and providing advice? | 0% (0/0) | 100% (7/7) | 7 |  |
| Functionality | Which method do you feel lead to the most mistakes? | 11.1% (1/9) | 88.9% (8/9) | 9 |  |
| *N: number of CHWs who answered* | | | | |  |
