# Supplementary material for: Development and evaluation of a mobile application for case management of small and sick newborns in Bangladesh
Source: BMC Med Inform Decis Mak. 2019 Jun 20;19:116. doi: 10.1186/s12911-019-0835-7 (PMC6585142; doi:10.1186/s12911-019-0835-7)
Supplement: Supplementary file 8 — Table S5. CHW Survey Responses about mCNCP Interface and Features. Results summarizing community health workers’ (CHWs') opinions of the simplicity, functionality, and usability of mCNCP’s interface and features. (DOCX 16 kb) [file 12911_2019_835_MOESM8_ESM.docx]

|  | | | | |
| --- | --- | --- | --- | --- |
| **Theme** | **Question** | **Agree/ Yes** | **Disagree/ No** | **N** |
| Simplicity | Are the questions easy to understand in Bangla? | 100% (12/12) | 0% (0/12) | 12 |
| Simplicity | Is the Bangla font legible on the app? | 100% (12/12) | 0% (0/12) | 12 |
| Simplicity | Are you able to read the captions on the pictures: for male or female? | 100% (12/12) | 0% (0/12) | 12 |
| Simplicity | Are you able to read the other captions? | 100% (10/10) | 0% (0/10) | 10 |
| Simplicity | Did you find the pictures and diagrams in the app clear and easy to use? | 100% (12/12) | 0% (0/12) | 12 |
| Functionality | Were you able to correct any errors you made during the newborn assessment using the app? | 100% (10/10) | 0% (0/10) | 10 |
| Usability | Are you able to press ALL of the buttons on the app with ease? | 100% (12/12) | 0% (0/12) | 12 |
| Usability | Was it easy for you to 'swipe' back to previous pages to make corrections? | 100% (12/12) | 0% (0/12) | 12 |
| Usability | *Did you have problems navigating the app: swiping to move to the next page (or screen) of the app?* | 16.7% (2/12) | 83.3% (10/12) | 12 |
| Usability | *Did you have problems navigating the app: scrolling to view the whole summary page (or other pages)?* | 18.2% (2/11) | 81.8% (9/11) | 11 |
|  |  | **Simplified** | **Medical** | **N** |
| Simplicity | Should the language of the questions be more formal (medical terms) or do you prefer simplified translations? | 100% (12/12) | 0% (0/12) | 12 |
|  |  | **Next Button** | **Swipe** | **N** |
| Usability | Do you prefer the 'swiping' function to move to the next page, or would you prefer a "Next" button to move to the next page? | 58.3% (7/12) | 41.7% (5/12) | 12 |
| *N: number of CHWs who answered* | | | | |
